# Evidence of nest material kleptoparasitism in Worm‐eating Warblers (*Helmitheros vermivorum*) in east‐central Arkansas, USA

**DOI:** 10.1002/ece3.7339

**Published:** 2021-04-09

**Authors:** Amy L. Wynia, James C. Bednarz

**Affiliations:** ^1^ Department of Biological Sciences Arkansas State University Jonesboro AR USA

**Keywords:** bottomland hardwood forests, neotropical migrant, stealing behavior, video camera systems

## Abstract

Nest material kleptoparasitism likely evolved in birds to reduce the cost of searching for and collecting material themselves. Although nest material kleptoparasitism has been reported commonly in colonially nesting species, reports for solitary breeding species are infrequent, especially for neotropical migratory species. Here, we report potential and actual nest material kleptoparasitism in the Worm‐eating Warbler (*Helmitheros vermivorum*). We deployed video camera systems at passerine nests (*n* = 81) in east‐central Arkansas during summers 2011–2012. In one video, we observed a Worm‐eating Warbler stealing nesting material from a Hooded Warbler (*Setophaga citrina*) nest. One day later, we later observed a Worm‐eating Warbler landing within 0.5 m of the same warbler nest when the female was incubating, which possibly deterred a second theft of nesting material. In a third video recording, we observed another Worm‐eating Warbler landing within 1 m of an Indigo Bunting (*Passerina cyanea*) nest. As far as we could determine, neither of these latter two nest visits resulted in nest material kleptoparasitism. Potential benefits of nest material kleptoparasitism include reduced competition for limited nest materials, easy access to suitable material, reduced travel distance, and reduction of nest predation risk; however, costs include risk of attack by host or introducing parasites to one's nest. Importantly, this behavior could ultimately affect the behavioral and life history evolution of a species. We suggest further work should be conducted to determine the prevalence of nest material kleptoparasitism in Worm‐eating Warblers and other solitary breeding passerines, including efforts to quantify the benefits and costs of this behavior.

## Graphical abstract

We observed potential and actual nest material kleptoparasitism in the Worm‐eating Warbler (*Helmitheros vermivorum*) in east‐central Arkansas. On video, we observed one Worm‐eating Warbler stealing nesting material from a Hooded Warbler (*Setophaga citrina*) nest and another landing within 1 m of an Indigo Bunting (*Passerina cyanea*) nest. Importantly, this behavior could ultimately affect the behavioral and life history evolution of a species.

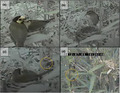

## 1 INTRODUCTION

Avian behavior affects individual fitness, reproductive success, and ultimately shapes the evolved life history of a species. For example, Lack (1968) suggested avian offspring exhibit developmental adaptations that are driven by strong selective pressures. These pressures include depredation risk and food availability (Sibly et al. 2012). These adaptations theoretically increase fitness and reproductive success and enable individuals to cope with various ecological challenges during the breeding season. One challenging process that likely increases the exposure and risk to nest depredation, is time‐consuming, and energetically costly for adults is nest construction (Mainwaring & Hartley, 2013 and references therein). During this period, birds must collect suitable nest material, which may be arduous to gather either because material is located far from the individual's nest or pair's territory, rare (Slager et al. 2012), or perhaps needed in large quantities that are difficult to collect (Jones et al. 2007).

Importantly, a trade‐off exists between the amount of time a bird invests in nest‐building and later reproductive stages (i.e., incubation and brood rearing). For example, Moreno et al. (2010) demonstrated females that collected less nest material spent more time incubating eggs and feeding nestlings, resulting in improved nestling growth. Further, research has demonstrated nest construction may play a role in sexual selection and in resulting reproductive success (Mainwaring & Hartley, 2013 and references therein). Moreover, Jones et al. (2007) emphasized kleptoparasitism of nest materials may reduce the number of trips to a nest during construction, which could reveal the location of the nest to a brood parasite or predator. Thus, birds may avoid the cost of searching for dispersed and hard‐to‐find material if they can gather relatively large quantities of suitable material from a nest of another nearby individual (either intra‐ or interspecific); albeit the thief must locate a suitable nest first. This nest material kleptoparasitism ultimately may influence the evolution of a species’ life history and our understanding of a species’ reproductive investment (Mainwaring & Hartley, 2013 and references therein).

Nest material kleptoparasitism is frequently reported in colonial‐nesting birds that may engage in aggressive competition for nest materials (e.g., Moreno et al. 1995; Siegfried, 1971; Slager et al. 2012 and references therein). However, nest material kleptoparasitism in solitary breeding species (i.e., noncolonial‐nesting birds) is infrequently reported in the literature (but see Fulton, 2006; Jones et al. 2007; Lindell, 1996; Slager et al. 2012)—especially for neotropical migratory species. Because of the high avian species richness in the neotropics, Slager et al. (2012) found it surprising that this behavior has not been reported more frequently in this region. This lack of reporting both in the neotropics and with breeding migratory species in North America either may be due to available sampling methodology or it may be true biological phenomena, that is, occurs infrequently (Slager et al. 2012). Specifically, most previous reports of nest material kleptoparasitism (e.g., Jones et al. 2007) are based on opportunistic anecdotal observations at nests during the sensitive nesting period. In such cases, both the avian nest occupants and potential nest material kleptoparasites often will be wary of the presence of a human observer and will modify or interrupt natural behavior. Therefore, instances of stealing nest material may be severely under observed and under reported. Thus, nest material kleptoparasitism incidentally has been reported only for a few neotropical migrants breeding in the USA (e.g., Jones et al. 2007), and we found no records on nest material kleptoparasitism for the Worm‐eating Warbler (*Helmitheros vermivorum*).

This monotypic, rather secretive species is a neotropical migrant that breeds in deciduous and mixed forests of eastern USA and winters in Central America (Vitz et al. 2020). Worm‐eating Warblers construct their nests on the forest floor; females use skeletonized leaves and moss (*Polytrichum* spp.) stems and may line their nests with white‐tailed deer (*Odocoileus virginianus*) hair, pine needles, fine grass, horsehair, or maple (*Acer* spp.) seed stems (Vitz et al. 2020 and references therein). Previous observations reported a Worm‐eating Warbler stole prey from another passerine (Kellner & Cooper, 1998); however, stealing nesting material has not been reported. To our knowledge, we present the first reported (and recorded) incidence of nest material kleptoparasitism by Worm‐eating Warblers.

## 2 METHODS

**FIGURE 1:**
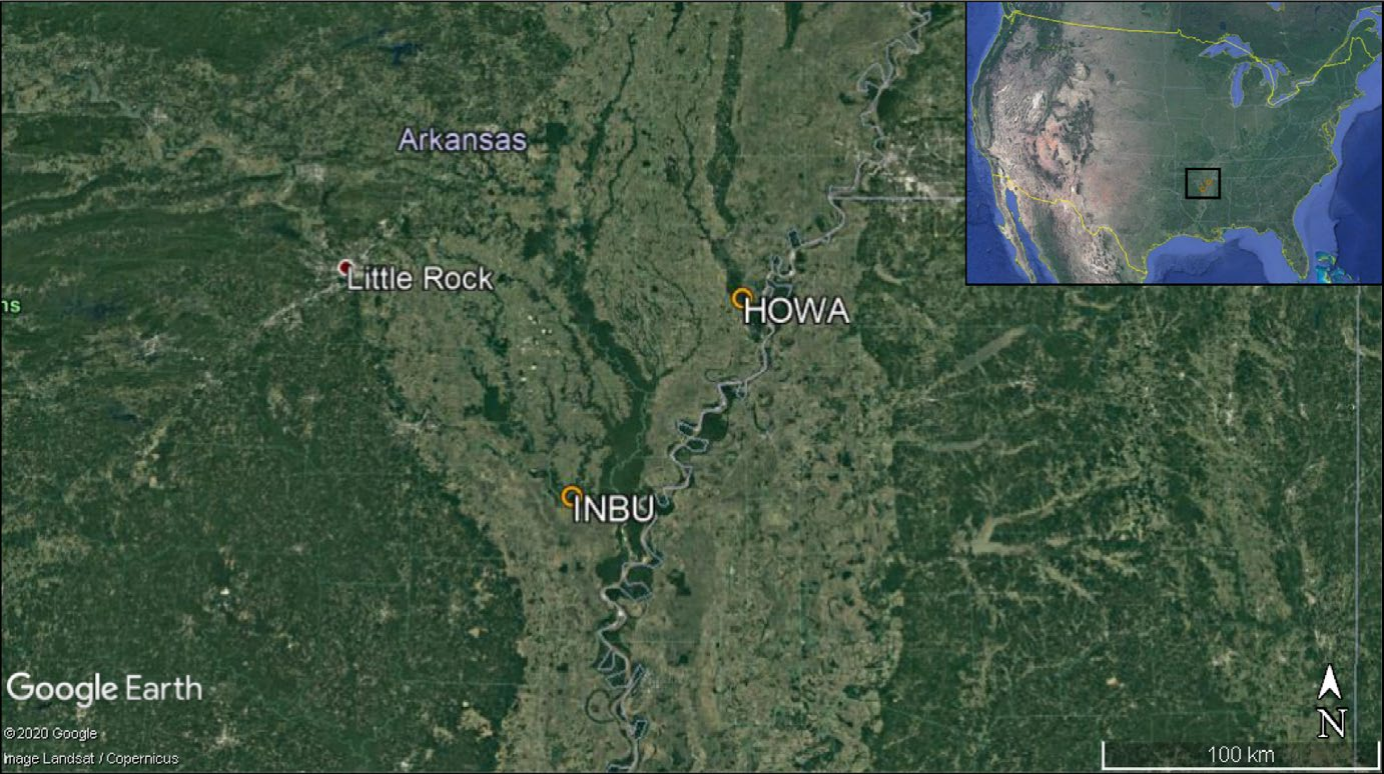
Study sites where we observed Worm‐eating Warblers (*Helmitheros vermivorum*) stealing or likely attempting to steal nest material from other passerine species’ nests during summers 2011–2012. INBU indicates an Indigo Bunting (*Passerina cyanea*) nest in Trusten Holder Wildlife Management Area (N 33° 59' 21.584", W 91° 20' 53.917") and HOWA indicates a Hooded Warbler (*Setophaga citrina*) nest in Saint Francis National Forest (N 34° 38' 44.840", W 90° 40' 9.974"). Both sites are in east‐central Arkansas, USA. Maps from Google Earth

We recorded our Worm‐eating Warbler observations while conducting an intensive study on the nesting ecology of passerines in bottomland hardwood forests. These observations were made at two study sites located in east‐central Arkansas, USA: Saint Francis National Forest (N 34° 38' 44.840", W 90° 40' 9.974") and Trusten Holder Wildlife Management Area (N 33° 59' 21.584", W 91° 20' 53.917") (Figure 1). Saint Francis is composed of over 8,500 ha of bottomland and upland forests (Benson et al. 2009) and Trusten Holder is comprised of over 4,000 ha of bottomland hardwood forest (AGFC 2020). In the larger research project (i.e., the intensive nesting ecology study), we established an approximately 72‐ha grid at each site. We conducted nest searches for all understory passerine species from early May through late July 2011–2012 and deployed video camera systems recording 24 hr/day at 81 nests of 10 different species. Camera systems consisted of a Supercircuits mono‐power infrared camera (PC177IR‐1color, Liberty Hill, TX), a micro‐digital video recorder (DVR; AKR‐100S, Korea), and a 12‐V deep‐cycle marine battery. We reviewed approximately 13,500 hr (i.e., 562.5 days) of video with camera systems deployed between 1–13 days per nest.

## 3 RESULTS

**FIGURE 2:**
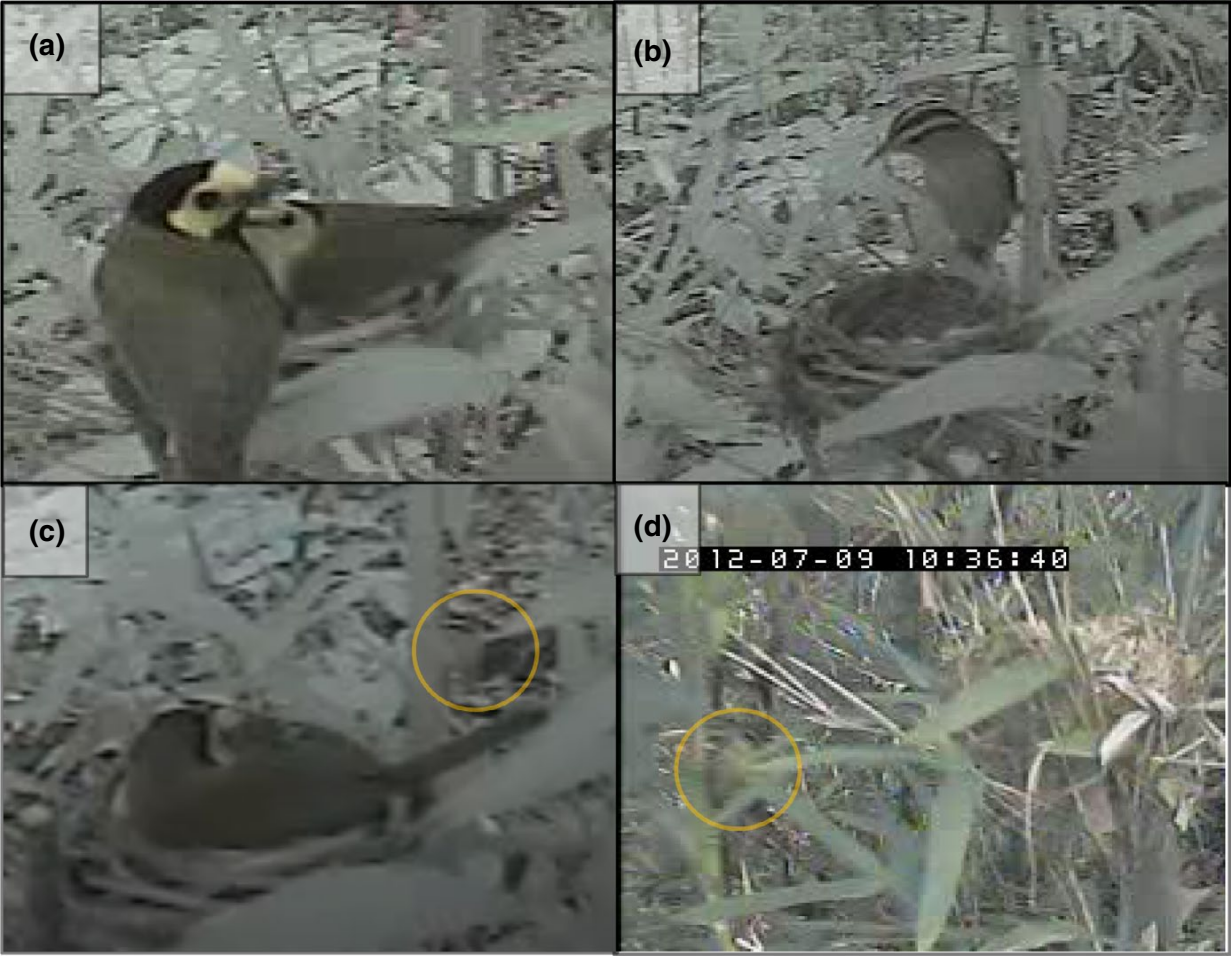
(a) Hooded Warblers (*Setophaga citrina*; male left, female right) on their nest on 21 June 2011 at 09:23:06 in Saint Francis National Forest (N 34° 38′ 41.7′′, W 90° 40′ 3.5′′) in east‐central Arkansas, USA. (b) Worm‐eating Warbler (*Helmitheros vermivorum*) stealing nest material from the Hooded Warblers’ nest nearly 9 hr later at 18:02:08. (c) Female Hooded Warbler on nest on 22 June 2011 with Worm‐eating Warbler in background (circled in orange). (d) Worm‐eating Warbler near an Indigo Bunting (*Passerina cyanea*) nest (N 33° 59′ 4.8′′, W 91° 20′ 45.6′′) in Trusten Holder Wildlife Management Area, east‐central Arkansas, USA

We observed three occasions where Worm‐eating Warblers visited other passerines’ nests (Figure 2). On 21 June 2011, a female Hooded Warbler (*Setophaga citrina*) left her nest (N 34° 38′ 41.7′′, W 90° 40′ 3.5′′, Figure 1) at 17:59:03 and nearly 3 min later, a Worm‐eating Warbler landed on a branch at 18:01:58 within 1 m of the nest. This nest was in river cane (*Arundinaria gigantea*) approximately 1 m from the ground and contained one host egg and three Brown‐headed Cowbird (*Molothrus ater*) eggs. The Worm‐eating Warbler flew to another branch closer to the nest and then landed on the nest at 18:02:05. At 18:02:08, the warbler began collecting nest material from the rim and inside cup of the nest—vigorously at times—and left with a bill‐full of material at 18:03:09. The female Hooded Warbler returned to the nest 7 min later at 18:10:37 and began fixing the out of place nest material at 18:10:49 before resuming incubation (Video 1).

**VIDEO 1:** Video of a Worm‐eating Warbler (*Helmitheros vermivorum*) stealing nesting material from a Hooded Warbler (*Setophaga citrina*) nest in east‐central Arkansas, USA on 21 June 2011 at 18:02:08. Note ~ 7 min are cut between the thief leaving and the victim returning. Video content can be viewed at https://onlinelibrary.wiley.com/doi/10.1002/ece3.7339

On the second occasion the following day (22 June 2011), a Worm‐eating Warbler (likely the same bird) appeared on a branch at 07:27:23 within 1 m of the same warbler nest; however, the female Hooded Warbler was on her nest incubating eggs. The Worm‐eating Warbler flew to a branch within 0.5 m of the incubating Hooded Warbler at 07:27:34, back to its original branch at 07:27:37, and flew off frame at 07:27:43. The Hooded Warbler seemed to be watching the Worm‐eating Warbler during this entire visit; however, she never moved from her nest (Video 2). The Worm‐eating Warbler did not steal any nesting material on this occasion and was not seen during the remainder of the video recording (i.e., 7 days later when all four chicks fell out of a rain‐saturated nest during a storm).

**VIDEO 2:** Video of a Worm‐eating Warbler (*Helmitheros vermivorum*) likely attempting to steal nesting material from a Hooded Warbler (*Setophaga citrina*) nest in east‐central Arkansas, USA on 22 June 2011 at 07:27:23. Video content can be viewed at https://onlinelibrary.wiley.com/doi/10.1002/ece3.7339

The third occasion occurred on 9 July 2012. A female Indigo Bunting (*Passerina cyanea*) flew from her nest (N 33° 59′ 4.8′′, W 91° 20′ 45.6′′, Figure 1) at 10:32:09; approximately 4 min later, a Worm‐eating Warbler landed at 10:36:38 in cane within 1 m of the nest (Figure 2). This nest was in river cane approximately 1.5 m from the ground and contained a 7‐day old Indigo Bunting chick. The Worm‐eating Warbler flew away 4 s later (10:36:42); it did not appear to take any nesting material and was not seen on video for the remainder of the recording (i.e., two days later when the chick fledged). The female Indigo Bunting returned to feed her nestling 1 min after the warbler left (i.e., 10:37:46); however, no buntings were seen on video during the duration of the warbler's visit.

## 4 DISCUSSION

The evolution of nest material kleptoparasitism may have several adaptive advantages including reduced time and effort in searching for and gathering material for the kleptoparasite (Jones et al. 2007), easy access to suitable material, reduced travel distance, and reduced competition for limited supply of materials (Slager et al. 2012). Thus, by stealing nest material individuals may spend more time defending nests, laying eggs (Jones et al. 2007), constructing nests, caring for young (Mainwaring & Hartley, 2013 and references therein), and foraging. Moreover, individuals may reduce depredation risk by not descending to the ground to collect material from the forest strata or by spending less time in less familiar territory (Jones et al. 2007; Slager et al. 2012). Additionally, depredation and brood parasitism risk may be alleviated by individuals taking shorter flights to pirate material or making fewer visits to collect material (Jones et al. 2007; Ley et al. 1997).

There are risks, however, to stealing material; hosts may defend their potentially valuable nest material and attack kleptoparasites, causing injury or perhaps death, or nest parasites may be transmitted to the kleptoparasite's nest (Jones et al. 2007; Ley et al. 1997). Yet, in other studies and likely in ours, kleptoparasites returned to the same nests to steal material on multiple occasions, suggesting the benefits of kleptoparasitism, at least in some circumstances, outweigh the costs (Jones et al. 2007; Slager et al. 2012).

Nest material kleptoparasitism occurs within passerine species—including warblers. For example, Jones et al. (2007) observed Cerulean Warblers (*Setophaga cerulea*) pirating nest material on multiple occasions from other warblers and other passerines as well. These researchers also reported anecdotes of kleptoparasitism by American Redstarts (*Setophaga ruticilla*), a Black‐throated Green Warbler (*Setophaga virens*), and a Northern Parula (*Setophaga americana*). Other passerines documented as kleptoparasites include Blue‐gray Gnatcatchers (*Polioptila caerulea*), Orchard Orioles (*Icterus spurius*), and Red‐eyed Vireos (*Vireo olivaceus*) (Jones et al. 2007). All these observed kleptoparasitism incidences were interspecific.

Here we document actual and potential nest material kleptoparasitism in another warbler species—the Worm‐eating Warbler. Notably, this species is relatively uncommon in our study area (USFWS 2020). In fact, in our larger avian breeding ecology study, we found 282 active passerine nests in the forests’ lower strata, but not one Worm‐eating Warbler nest. Thus, these observations suggest nest material kleptoparasitism may be relatively common in Worm‐eating Warblers at least in our study areas.

All three of our actual or potential nest material kleptoparasitism events were interspecific as well. Interestingly, our observations suggest the Worm‐eating Warblers were not always successful in stealing nesting material. The incubating Hooded Warbler prevented the Worm‐eating Warbler from stealing more material, although it was not for a lack of trying. The short duration of the third incident (i.e., 4 s) could indicate an Indigo Bunting parent may have been present nearby off camera, deterring this Worm‐eating Warbler from taking nesting material. The female bunting returned to her nest within 1 min of the warbler departing; therefore, it may have been in the vicinity and startled or chased the warbler off camera. As our video did not record audio, we cannot ascertain if any buntings were chipping (i.e., vocalizing warnings) nearby. Further, the bunting video resolution was limited; thus, we could not determine if the warbler already had nest material in its bill. We also cannot rule out that the warbler landed near this nest for another purpose unrelated to nest material kleptoparasitism.

We suggest the host species of the victim nest may influence the likelihood of nest material kleptoparasitism by Worm‐eating Warblers and other kleptoparasitic species. For example, Hooded Warblers are likely incapable of inflicting a serious or fatal wound on another species of nest material‐stealing warbler. However, the Northern Cardinal (*Cardinalis cardinalis*) with its heavy seed‐crushing bill could inflict serious damage on a warbler nest material thief. Further, avian species use various nesting materials; therefore, victims selected for nest material theft likely are limited. Specifically, Worm‐eating Warblers use relatively delicate nesting material such as fine grass, mammalian hair, and moss compared to more coarse nesting material such as heavy grasses and small sticks used by Northern Cardinals. For these reasons, we predict Worm‐eating Warblers would unlikely attempt to parasitize materials from a Northern Cardinal nest.

Notably, our sample size was small and limited to individuals inhabiting two forested regions in east‐central Arkansas; therefore, we encourage researchers to examine whether this nest material kleptoparasitism behavior exists in other populations throughout the Worm‐eating Warbler's range. Further, given nest material kleptoparasitism is infrequently reported in the literature, we encourage researchers to investigate this behavior throughout the noncolonial breeding avian community to determine if it is indeed more widespread than presently recognized. With the advent of remote nest camera technology, as used in our study, we suggest more systematic and accurate assessments can be made on the incidence and costs and benefits of nest material kleptoparasitism. Specifically, to study this phenomenon nest cameras might be installed during the nest‐building stage and after nestlings are depredated or fledged. Importantly, quantification of behavior and aspects of natural history are necessary for the scientific community and managers to better understand the selective forces and factors influencing life history traits and population viability. Moreover, natural history studies are needed to increase public awareness, general knowledge, and scientific progress (Xiao et al. 2017 and references therein).

## CONFLICT OF INTEREST

None declared.

## AUTHOR CONTRIBUTIONS

AW and JB conceived the ideas and designed the methodology, JB secured funding, AW collected and extracted data from video recordings and led the writing of the manuscript. AW and JB contributed critically to drafts and gave final approval for publication.

## Data Availability

Original data and video pertaining to this research are available on the Dryad Digital Repository:
